# Growth in the Lower Limb Following Chemotherapy for a Malignant Primary Bone Tumour: A Straight-Line Graph

**DOI:** 10.1080/13577149778335

**Published:** 1997-06

**Authors:** Paul Cool, Mark Davies, Rob J. Grimer, Simon R. Carter, Roger M. Tillman

**Affiliations:** 1 The Royal Orthopaedic Hospital Oncology Service Birmingham UK; 2 Walnut Cottage Upton Magna Shropshire SY4 4TZ UK

## Abstract

*Purpose*. The aim of this paper was to assess the growth in the unaffected lower limb of children who had received chemotherapy for a malignant primary bone tumour around the knee.

*Subjects/methods*. Following diagnosis, all children (45, of which 32 were boys and 13 were girls) were staged. If limb-salvage surgery was thought appropriate, measured radiographs of both legs was performed, the bone age was estimated and the expected growth in the femur and tibia was calculated according to Tupman. These procedures were repeated at follow-up and the data plotted. Regression and correlation coefficients were also calculated.

*Results*. The observed regression line in boys was almost identical to Tupman's curve. However, the observed growth in girls was larger than the expected growth.

*Discussion*. It is recommended that the regression lines presented here are used in the calculation of the expected growth in the lower limb of children who have received chemotherapy for a malignant primary bone tumour, especially in girls.


*Sarcoma* (1997) **1**, 75–77

ORIGINAL ARTICLE

### Growth in the lower limb following chemotherapy for a malignant primary bone tumour: a straight-line graph

PAUL COOL, MARK DAVIES, ROB J. GRIMER, SIMON R. CARTER & ROGER M. TILLMAN

*The Royal Orthopaedic Hospital Oncology Service, Birmingham, UK*

**Abstract**
*Purpose.* The aim of this paper was to assess the growth in the unaffected lower limb of children who had received chemotherapy for a malignant primary bone tumour around the knee.
*Subjects/methods.* Following diagnosis, all children (45, of which 32 were boys and 13 were girls) were staged. If limb-salvage surgery was thought appropriate, measured radiographs of both legs was performed, the bone age was estimated and the expected growth in the femur and tibia was calculated according to Tupman. These procedures were repeated at follow-up and the data plotted. Regression and correlation coefficients were also calculated.
*Results.* The observed regression line in boys was almost identical to Tupman's curve. However, the observed growth in girls was larger than the expected growth.
*Discussion.* It is recommended that the regression lines presented here are used in the calculation of the expected growth in the lower limb of children who have received chemotherapy for a malignant primary bone tumour, especially in girls.

**Key words**: *bone tumour, chemotheraphy, growth.*

### Introduction

Neo-adjuvant chemotherapy has greatly improved the survival in children with malignant primary bone tumours. The overall survival in patients with osteosarcoma is nowadays 64%.[1]

Improvements in diagnostic imaging, surgical technique, biomedical engineering and survival have made limb-salvage surgery in children possible. One limb-salvage option for children with a malignant primary bone tumour around the knee is replacement with a custom-made extensible endoprosthetic replacement.[2–6] Our centre has performed extensible endoprosthetic replacements for primary bone tumours around the knee since 1976.

In order to manufacture the extensible endoprosthetic replacement, it is necessary to have an estimate of the expected growth in the lower limb. Our centre has used data provided by Tupman[7] to calculate this growth. However, these data are based on a normal population of children.

Fraumeni[8] reported that children with osteosarcoma are taller at the time of diagnosis than controls. However, others[9,10] did not find such a relation.

Glasser *et al.*[9] showed that children with malignant primary bone tumours had a marked retardation in growth during the year of cytotoxic chemotherapy. They concluded that final height might be affected, but to a small degree.

We could find no reports in the literature providing information about the growth in the lower limb of children who have received chemotherapy. The aim of this study was to assess the growth in the lower limb of children who had chemotherapy for a malignant primary bone tumour.

### Subjects and methods

Following the diagnosis of a malignant primary bone tumour of the distal femur or proximal tibia, all children were fully staged. This included a radiograph of the lesion, magnetic resonance imaging (MRI) or computed tomography (CT) of the lesion, a bone scan and a CT scan of the chest.

If the patient was thought to be suitable for limb-salvage surgery, measured radiographs of both legs were also performed. The bone age was also estimated according to the Greulich and Pyle method.[11] With the bone age, the expected growth in the femur and tibia was calculated according to Tupman.[7]

All patients received chemotherapy in a neo-adjuvant setting according to the then current

Correspondence to: W. P. Cool, Walnut Cottage, Upton Magna, Shropshire SY4 4TZ, UK. Tel: + 44 1691 404000 page 147 (daytime); + 44 1743 709210; E-mail: pcool@netcomuk.co.uk.

1357-714X/97/020075-03 $9.00 ©1997 Carfax Publishing Ltd

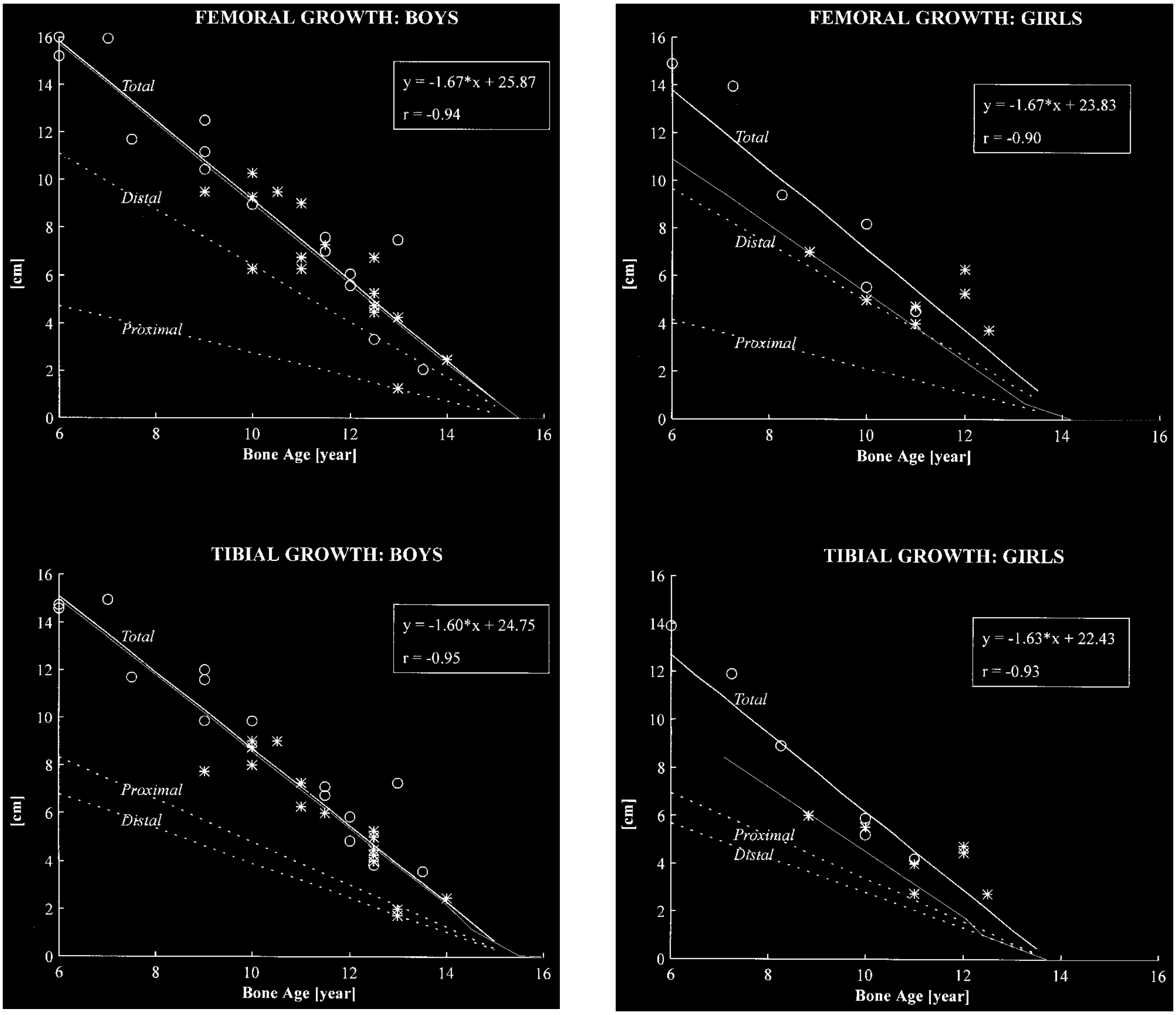

**Fig. 1.** *Growth at the latest time of follow-up in the femur and tibia in 45 children who received chemotherapy for a malignant primary bone tumour around the knee. Children who were skeletally mature at review are indicated by ✳ and children who were immature by ○. Tupman's curve is shown in grey. The dotted lines indicate the proximal and distal physeal growth as calculated from the regression line. The formula of the regression line and the correlation coefficient* (r) *are shown in the box.*

protocol. No patients received radiotherapy to the extremities. Limb-salvage surgery was performed and the patients were followed up in the outpatient clinic.

At the time of review, all patients had measured radiographs of both legs and their bone age estimated. The growth in the unaffected (normal) leg was calculated. As in Tupman's[7] review, the femur was measured from the top of the femoral head to the medial condyle. The tibia was measured from the top of the tibial spine to the tip of the medial malleolus.

Patients who were skeletally immature at review had the expected growth at the time of review added to the measured growth.

The data were plotted on a graph and superimposed on Tupman's[7] curve. The regression coefficients were calculated by means of the least square method and the regression line was plotted in the figure. Correlation coefficients were also calculated.

In order to plan limb-salvage surgery, it is extremely helpful to have an estimate of the expected growth in the proximal and distal physis of the affected bone. The growth in the proximal femoral physis has been reported[7] as 30% and the growth in the distal femoral physis as 70% of the total femoral growth. Similarly, the proximal tibial growth is 55% and the distal tibial growth 45% of the total growth in that bone.[7] With the aid of these figures, regression lines for the proximal and distal physeal growth in the femur and tibia were also calculated and added to the figure.

### Results

Between 1976 and 1992, our centre performed 106 extensible endoprosthetic replacements in children with a malignant primary bone tumour around the knee. Of these patients, 39 died.

Patients were excluded if the follow-up was less than 1.5 years or if insufficient radiographs were available to assess the growth. These criteria left 45 children in the study. There were 32 boys and 13 girls. The chronological age at diagnosis was on average 11.2 years (range 6.5–15.3 years) and the mean bone age was 10.6 years (range 6–14 years). All patients were fully staged as described earlier and received neo-adjuvant chemotherapy according to the relevant treatment protocol. In 37 cases the diagnosis was osteosarcoma, in seven cases, Ewing's sarcoma and malignant fibrous histiocytoma in the remaining patient. There were 29 patients who had a distal femoral tumour. The remaining 16 patients had a proximal tibial tumour. The left side was affected in 23 cases and the right side in 22 cases. The average follow-up was 4.5 years in both boys (range 2.1–7.9 years) and girls (range 1.7–11.1 years). On average, the chronological age at follow-up was 16.0 years in boys (range 12.4–20.8 years) and 14.7 years in girls (range 11.1–22.8 years).

Figure 1 shows the femoral and tibial growth that had occurred in boys and girls at the latest follow-up plotted against the bone age at diagnosis. There were two boys who had identical measurements, which explains why only 31 points are visible in the graphs for boys.

There were 17 boys (53%) and seven girls (54%) who were skeletally mature at latest time of follow-up (indicated by ✳); the remaining 21 children were skeletally immature (indicated by ○).

The regression lines and correlation coefficients are shown in the figure. Furthermore, regression lines for the proximal and distal growth in the femur and tibia were calculated as described earlier and added to the figure. Tupman's[7] original curve is also shown.

### Discussion

The observed regression line in boys is almost identical to Tupman's[7] curve. However, in girls the observed growth is larger than the expected growth. Although there were considerably less girls than boys in this study, virtually all girls had a growth that was equal to or larger than expected. Furthermore, the correlation coefficients are high. It seems therefore likely that the regression line in girls is a realistic indicator of growth. The reason for the sex difference remains unclear.

Tupman[7] performed his study in 1962. Therefore, it is possible that his data are out of date. Unfortunately, more recent data are not available. However, comparison with our regression lines indicates that growth following chemotherapy is independent of skeletal maturity prior to chemotherapy treatment. Furthermore, there does not seem to be a longlasting effect of chemotherapy on longitudinal growth in the lower limb.

A longer follow-up time is unlikely to influence the observed regression lines significantly, since 53% of the children were skeletally mature at the time of review and the correlation coefficients are high.

In conclusion, we recommend that these new regression lines are used in the calculation of the expected growth in the lower limb in children who received cytotoxic chemotherapy for a malignant primary bone tumour. This seems especially important in girls.

### References

1 Bramwell VHC, Burgers M, Sneath R, *et al.* A comparison of two short intensive adjuvant chemotherapy regimens in operable osteosarcoma of limbs in children and young adults: the first study of the European osteosarcoma intergroup. *J Clin Oncol* 1992; 10:1579–91.

2 Cool WP, Grimer RJ, Carter SR. Growth in distal femoral endoprosthetic replacements for the skeletal immature. *J Bone Joint Surg* 1995; 77B Suppl. I:86.

3 Cool WP, Grimer RJ, Carter SR, *et al.* The outcome of extensible endoprosthetic replacements of the proximal tibia. *J Bone Joint Surg* 1995; 77B Suppl. 3:331.

4 Scales JT, Sneath RS. The extending prosthesis. In: Coombs R, Friedlandler G, eds. *Bone tumour management*. London: Butterworths, 1987; 168–77.

5 Scales JT, Sneath RS, Wright KWJ. Design and clinical use of extending prostheses. In: Enneking WF, ed. *Limb salvage in musculoskeletal oncology*. New York: Churchill Livingstone, 1987: 52–61.

6 Sneath RS, Carter SR, Grimer RJ. Growing endoprosthetic replacements for malignant tumours. In: Langlais F, Tomeno B, eds. *Limb salvage—major reconstructions in oncologic and nontumoral conditions*. Berlin: Springer-Verlag, 1991; 573–8.

7 Tupman GS. A study of bone growth in normal children and its relationship to skeletal maturation. *J Bone Joint Surg* 1962; 44B:42–67.

8 Fraumeni JF. Stature and malignant tumors of bone in childhood and adolescence. *Cancer* 1967; 20:967–73.

9 Glasser DB, Duane K, Lane JM, *et al.* The effect of chemotherapy on growth in the skeletally immature individual. *Clin Orthop* 1991; 262: 93–100.

10 Operskalski EA, Preston-Martin S, Henderson BE, *et al.* A case-control study of osteosarcoma in young persons. *Am J Epidemiol* 1987; 126:118–26.

11 Greulich WW, Pyle SI. *Radiographic atlas of skeletal development of the hand and wrist*. 2nd edn. Stanford: Stanford University Press, 1966.
